# Subconjunctival Triamcinolone Acetonide in the Treatment of Non-necrotizing, Non-infectious Anterior Scleritis

**DOI:** 10.7759/cureus.94827

**Published:** 2025-10-17

**Authors:** Shreyas Temkar, Aahan D Shah, Hemanth Ramachandar, Amit K Deb

**Affiliations:** 1 Ophthalmology, Jawaharlal Institute of Postgraduate Medical Education and Research, Puducherry, IND

**Keywords:** anterior scleritis, immunosuppression, ocular inflammation, subconjunctival injection, triamcinolone acetonide

## Abstract

Introduction: Non-necrotizing, non-infectious anterior scleritis often requires systemic corticosteroids or immunosuppressants, which carry significant adverse effects. Local therapy in the form of subconjunctival triamcinolone acetonide injections (STI) can be beneficial in such patients.

Methods: This retrospective case series included six eyes of six patients treated with STI. Patients underwent detailed ocular and systemic evaluation prior to injection. Under peribulbar anesthesia, 2-4 mg of preservative-free triamcinolone acetonide was injected subconjunctivally at the site of inflammation. Patients were monitored for pain relief, congestion, intraocular pressure (IOP), cataract progression, and scleral necrosis.

Results: Four patients had diffuse and two had nodular scleritis. All patients achieved symptomatic relief and reduction in inflammation within one to two weeks. All patients were able to reduce or discontinue systemic immunosuppressive therapy. The mean symptom-free interval was 4.5 months, and the mean follow-up duration was 12.3 months. Complications included transient ocular hypertension in one patient and cataract progression in another. No cases of scleral necrosis were observed.

Conclusion: STI provides effective local disease control and symptom relief in non-necrotizing, non-infectious anterior scleritis, while reducing systemic treatment burden. They appear to be a safe, cost-effective adjunct, though careful patient selection and monitoring for IOP elevation are essential.

## Introduction

Scleritis encompasses a group of diseases marked by scleral inflammation, potentially resulting from local or systemic immune-mediated conditions or infections [1]. They are classified into anterior and posterior subtypes with the equator of the eye as an arbitrary division line [2]. Anterior scleritis is further divided into necrotizing and non-necrotizing types. Anterior scleritis often presents with significant pain and redness [3]. Prompt and effective treatment is crucial to provide adequate pain relief and prevent irreversible structural damage and vision loss. Nearly 60% of the patients require oral corticosteroids or immunosuppressive drugs for satisfactory disease remission [4]. However, these systemic therapies carry a significant risk of potential adverse effects. A few patients may also have relapses despite being on systemic treatment. Local therapy, which is commonly employed in the treatment of non-infectious uveitis, has not been widely accepted in the management of scleritis. The use of periocular steroids was previously considered to have an increased risk of scleral necrosis and perforation [5,6]. However, recent publications have shown them to be safe and effective without much risk of thinning and perforation [7-12]. Studies on the use of local therapy in the management of scleritis from Asia/India are particularly lacking in the literature. Encouraged by the available studies, we analyzed our results on the efficacy and safety profile of anterior subconjunctival triamcinolone acetonide injections (STI) in non-infectious, non-necrotizing anterior scleritis.

## Materials and methods

This was a retrospective analysis of the medical records of six patients diagnosed with non-infectious, non-necrotizing anterior scleritis treated with STI who had either adverse effects or had an inadequate response to systemic therapy. The study adhered to the Declaration of Helsinki. Patients with the necrotizing variant, preexisting scleral thinning, a history of glaucoma, and steroid responsiveness were excluded. All the patients had a careful ocular examination and systemic review to rule out infectious causes. Written informed consent was obtained from all the study participants before the injection procedure. Since the injection through an inflamed conjunctiva can be extremely painful, all the injections were done under a peribulbar block (~3 mL) to achieve adequate local analgesia. Topical proparacaine 0.5% was instilled to enhance the effect of anesthesia. The procedure was done under a surgical microscope to enhance visualization and prevent inadvertent scleral penetration of the needle. Preservative-free triamcinolone acetonide 40 mg/mL (Aurocort, Aurolab, India) was loaded in a 1 mL syringe and attached with a 30G needle. The freely mobile conjunctiva adjacent to the scleritis area was grasped and tented up using atraumatic conjunctival forceps, and the needle was advanced carefully toward the center of the lesion. Around 0.05 to 0.1 mL (2 to 4 mg) of triamcinolone acetonide solution was injected at one to two locations over the area of scleral inflammation. A cotton-tipped applicator was used to control the blood ooze at the site of conjunctival entry. A video demonstrating the surgical technique is being provided (Video 1). Patients were asked to continue their systemic medications, which were subsequently modified based on response to STI. At each visit, patients were assessed in terms of pain relief, severity of congestion, intraocular pressure (IOP) rise, cataract development, and any evidence of scleral necrosis.

**Video 1:** Subconjunctival triamcinolone acetonide injection for anterior scleritis.

## Results

The mean age of the patients was 48.2 years. Among the six patients, five were females (83%) and one was a male patient (17%). Bilateral disease was observed in one patient (17%), whereas the other five showed unilateral involvement (83%). One male patient (patient 2) and four female patients (patients 1, 3, 4, and 6) had unilateral disease. One female patient (patient 5) had bilateral scleritis and received the injection only in one eye with resistant disease. Patient characteristics are listed in Table 1. Common underlying systemic associations noted were diabetes in two of the patients, and four of them had complications from systemic steroid administration. Four patients had diffuse scleritis (67%), whereas the other two presented with nodular scleritis (33%). The mean duration of disease before injection was 11.6 months, and the mean follow-up period was 12.3 months. The clinical features of the patients included in this study are outlined in Table 1.

**Table 1:** Clinical details of patients with anterior scleritis receiving subconjunctival triamcinolone acetonide injections.

Patient no.	Age (in years)	Gender	Scleritis type	Pre-injection systemic treatment (per day dose)	Associated systemic comorbidities	Duration of scleritis pre-injection (in months)	Systemic treatment post-injection	Symptom-free interval post-injection	Total number of injections	Duration of follow-up	Local side effects
01	49	Female	Non-necrotizing nodular	Prednisolone 60 mg, mycophenolate mofetil 1000 mg	Steroid-induced cushing syndrome and hyperglycemia	08	Prednisolone 5 mg per day, mycophenolate mofetil 2000 mg per day	06	03	14	Cataract progression
02	69	Male	Non-necrotizing diffuse	Prednisolone 60 mg	Diabetes mellitus	36	Nil	03	01	03	Nil
03	41	Female	Non-necrotizing diffuse	Prednisolone 40 mg	Osteoporosis, cortisol deficiency, steroid-induced hyperglycemia	06	Prednisolone 10 mg per day	08	01	08	Nil
04	38	Female	Non-necrotizing diffuse	Prednisolone 50 mg	Steroid-induced hyperglycemia	07	Prednisolone 10 mg per day, methotrexate 15 mg once weekly	01	02	19	Ocular hypertension
05	44	Female	Non-necrotizing diffuse Bilateral	Prednisolone 60 mg	Reactive airway disease, steroid-induced hyperglycemia	12	Nil	05	01	07	Nil
06	48	Female	Non-necrotizing nodular	Indomethacin 150 mg	Diabetes mellitus	01	Nil	04	01	04	Nil

**Figure 1:**
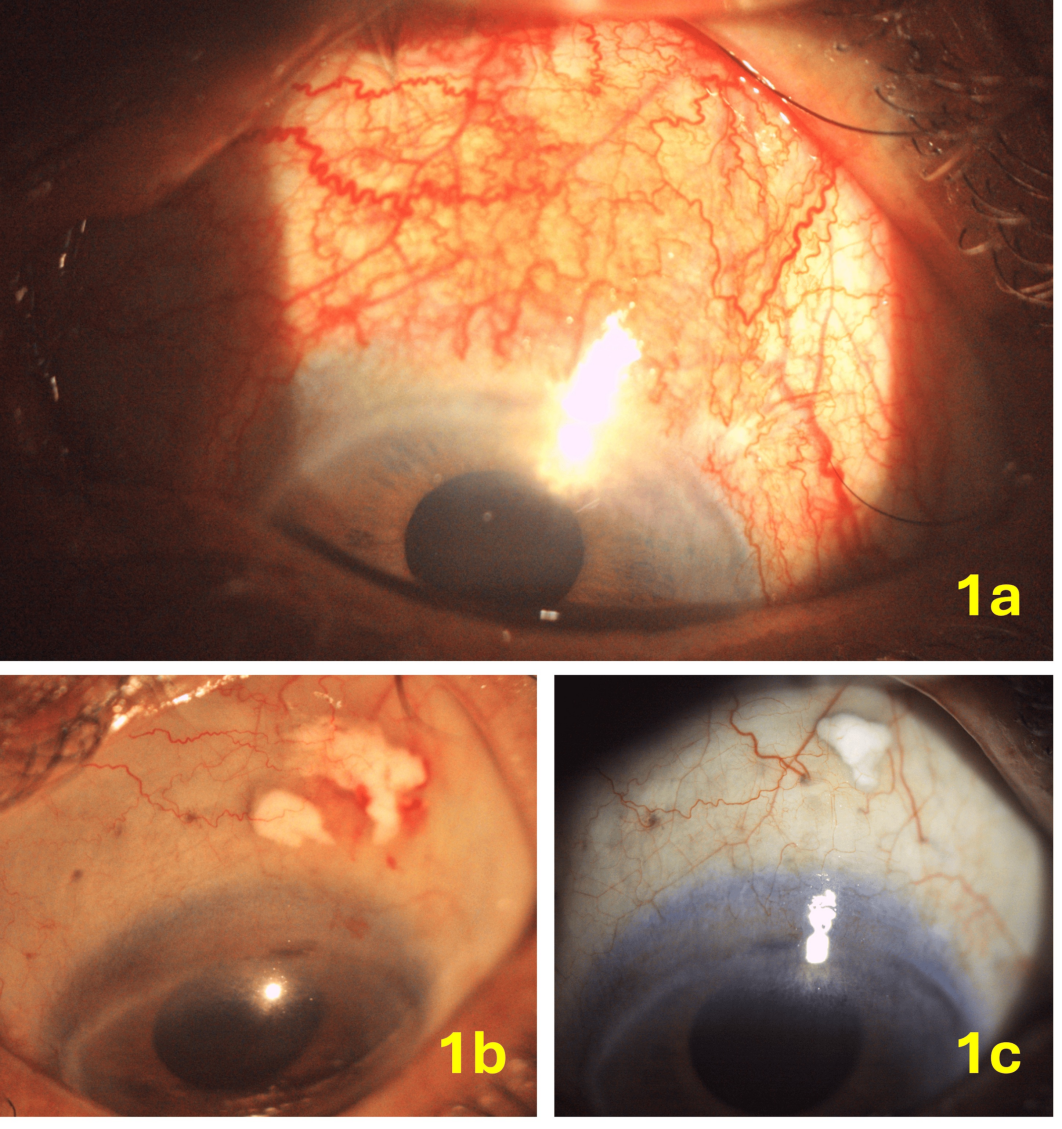
Patient 2 with diffuse anterior scleritis (1a); significant resolution of congestion after one week of anterior subconjunctival triamcinolone acetonide injection (1b); the patient remained symptom-free even after eight weeks of injection (1c).

**Figure 2:**
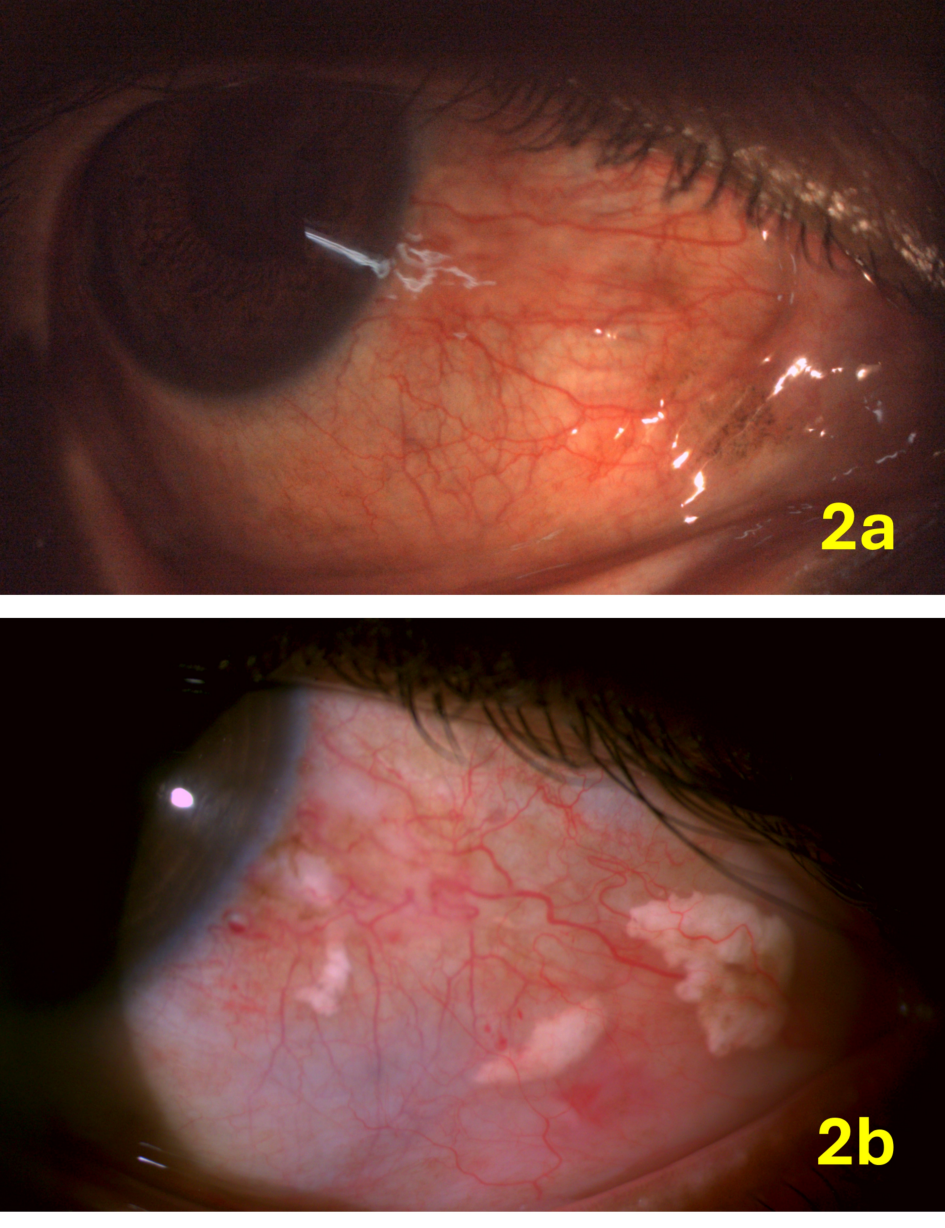
Patient 4 with diffuse scleritis confined to the temporal aspect (2a); two weeks after the injection, the scleritis activity is reduced, with visible subconjunctival triamcinolone deposits (2b).

**Figure 3:**
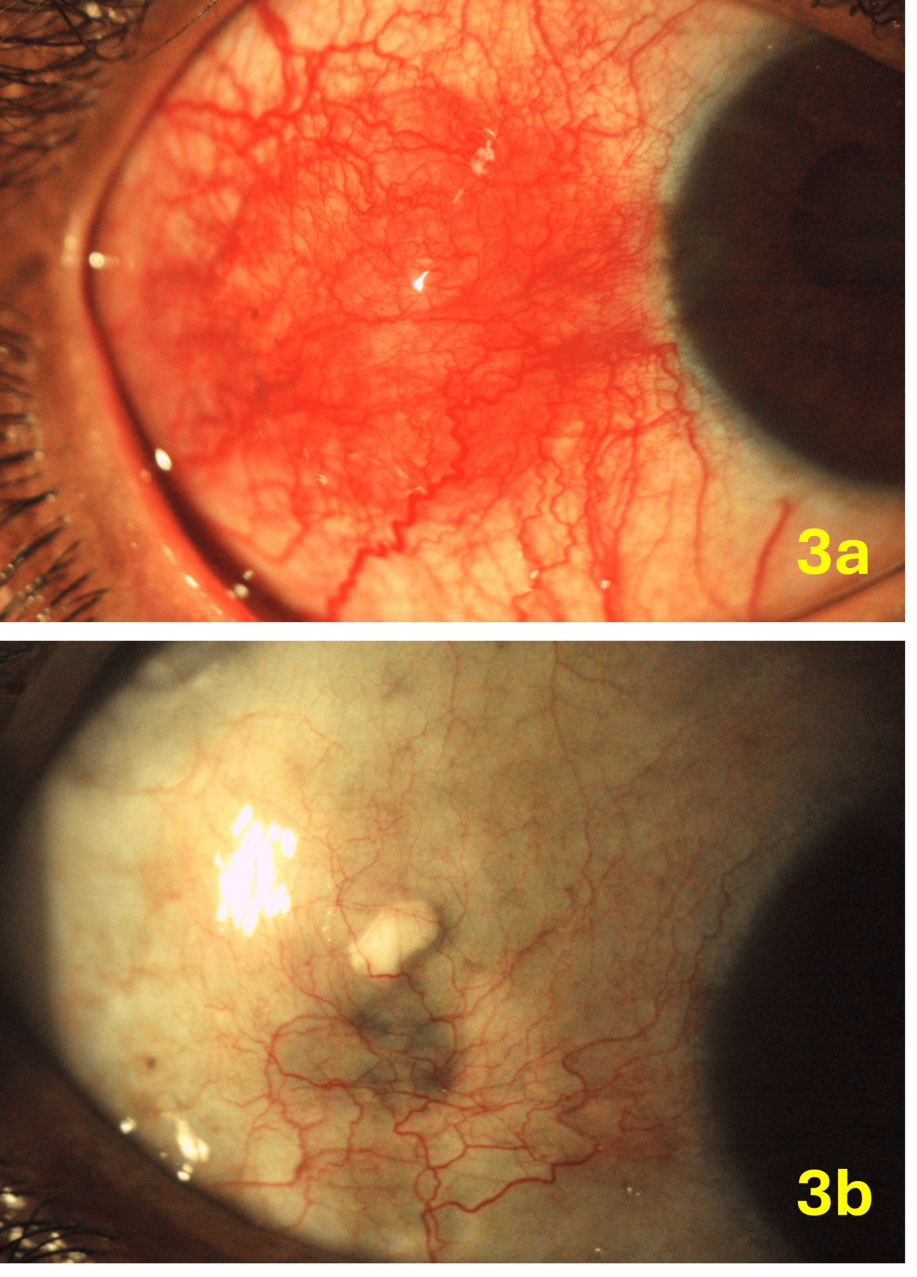
Patient 6 with nodular scleritis of the right eye (3a). Four weeks post-injection, the scleritis activity is markedly reduced (3b). Some scleral thinning is noted in the area corresponding to the center of the nodule, likely attributable to the disease being more severe in the center of the nodule, resulting in focal scleral thinning.

Local complications included cataract progression in one patient (patient 1) and ocular hypertension in another patient (patient 4), which resolved with topical anti-glaucoma medication usage. A small area of scleral thinning was noted on follow-up in one patient of nodular scleritis (patient 6). This is probably related to the healing response seen in nodular scleritis, which represents a more severe form of disease compared to the diffuse variant. None of the patients developed scleral necrosis at the site of injection. The average symptom-free interval post-injection was 4.5 months, with the average duration of follow-up being nine months. Representative pre- and post-injection images of three patients are provided in Figures 1-3.

Patient 1, maintained on 5 mg of prednisolone and 2000 mg of mycophenolate mofetil per day, required two additional depot injections at three to six-month intervals. She had developed bilateral posterior subcapsular cataracts (slightly more in the eye receiving injections). Successful phacoemulsification with intraocular lens implantation was performed in both eyes subsequently. Since the patient was on long-term systemic steroids, it is difficult to attribute the local steroid injection alone as a cause of cataract progression. Patient 4 required a repeat injection and reintroduction of oral steroids after two months. She developed an IOP rise (to 28 mmHg) three weeks after the second injection. It got well controlled (<20 mmHg) with topical timolol eye drops. Timolol was stopped after three months. Patients 2, 3, 5, and 6 did not require a second injection and remained disease-free during the follow-up period. It was interesting to note that recurrences noted in patients 1 and 4 were in a site different from the initial site/site where the injection was given.

## Discussion

Scleritis is a rare, vision-threatening inflammation of the sclera, often associated with potential systemic illnesses such as rheumatoid arthritis, granulomatosis with polyangiitis, and relapsing polychondritis. However, a significant proportion (nearly 2/3rd of patients) have isolated scleritis without any systemic inflammation [13]. Management of scleritis, whether or not associated with systemic diseases, often necessitates the use of systemic non-steroidal anti-inflammatory drugs, steroids, and immunosuppressants to control inflammation. In a comprehensive review, it was noted that nearly 60% of patients with scleritis require systemic therapy, and nearly 37.5% of them experience significant adversities related to the treatment [4]. Similarly, a small proportion of patients have persistent scleritis activity despite maximum immunosuppression. These situations necessitate the need for exploring other treatment options.

Local therapy has been very often utilized in the management of non-infectious uveitis. Posterior subtenon and intravitreal steroids provide significant control of local inflammation without having notable systemic side effects. Similar use of local steroid therapy is not well accepted in the management of scleritis. However, over the years, literature has established STI to be a safe and effective adjunctive treatment for resistant, non-infectious, non-necrotizing anterior scleritis [7-12]. This treatment modality aims to deliver high local concentrations of corticosteroids while minimizing systemic exposure. Our study similarly showed that all six patients had remission of disease activity, reduction in pain, and significant reduction in systemic treatment burden. Except for minor side effects like the rise in IOP in one patient and probable cataract progression in another patient, the STI proved to be a safe local treatment option. 

Recent retrospective studies extending over the last two decades have supported the efficacy and safety of periocular steroid injections. Early studies by Tu et al. (1995) and Croasdale and Brightbill (1999) demonstrated that use of subconjunctival corticosteroid injections for treating non-necrotizing anterior scleritis could achieve rapid symptomatic relief and disease resolution with minimal systemic exposure [7,8]. Subsequent studies by Zamir et al. (2002), Albini et al. (2005), and Roufas et al. (2010) confirmed the efficacy of subconjunctival triamcinolone acetonide, showing significant improvement in pain and inflammation with a favorable safety profile [9-12]. Sohn et al. conducted a multicenter retrospective study evaluating 68 eyes with non-necrotizing, non-infectious anterior scleritis treated with STI (2-8 mg). Over a median follow-up of 2.3 years, 97% of eyes showed improvement after a single injection, with more than half remaining recurrence-free at two to four years. Many patients were able to reduce or discontinue systemic corticosteroids or immunosuppressive agents, especially those without underlying systemic disease. The main adverse effect was ocular hypertension, occurring in about 20% of eyes, though only a few required medical or surgical intervention. Importantly, no cases of scleral melt or necrosis were observed. Table 2 summarizes the literature evaluating the utility of STI for anterior scleritis.

**Table 2:** Comparison of studies evaluating the utility of subconjunctival triamcinolone acetonide injections for anterior scleritis. IOP, intraocular pressure

No	Citation	Study design	Population (N)	Key results	Follow-up
1	Tu EY et al. [7]	Retrospective case series	20 patients	90% relief; 45% no further therapy; no scleral necrosis/perforation	≥6 weeks (average 18 weeks symptom-free)
2	Croasdale CR, Brightbill FS [8]	Small case series	8 patients	88% relief; 57% repeat injection; no scleral necrosis	Variable
3	Zamir E et al. [9]	Prospective case series	12 eyes (10 patients)	11/12 resolved; 6/10 stopped systemic therapy	Median 15 months
4	Albini TA et al. [10]	Retrospective case series	38 eyes (35 patients)	36/38 resolved; few IOP/cataract/glaucoma; no necrosis	Median 29 months
5	Roufas A et al. [11]	Retrospective case series	12 patients	23/25 resolved; 38% relapse; 4 had IOP rise	Mean 9 months (1-20 months)
6	Sohn EH et al. [12]	Retrospective multicenter	68 eyes (53 patients)	97% response after 1 injection; 68% remained relapse free at 24 months, 50% at 48 months; 20% developed ocular hypertension; no necrosis	Median 2.3 years (0.5-8.3 years)
7	Present study	Retrospective case series	6 patients	All improved; mean symptom-free interval 4.5 months; 2 needed repeat injection; 1 IOP rise (controlled), 1 cataract progression; no scleral necrosis	Mean 12.3 months (range 4-19 months)

Literature, as well as our study, has demonstrated STI to be a safe adjunct in the management of non-infectious, non-necrotizing anterior scleritis. Nevertheless, it is always imperative to keep a watch for potential adverse effects, including scleral necrosis/melt and possible localized infections [14]. Cautious patient selection, excluding necrotizing and infectious variants of scleritis, is a key to achieving success with this procedure without undue complications. 

This study has certain limitations. It was retrospective in nature, included a limited number of patients, and lacked a comparison group. Additionally, no objective methods were used to quantify changes in scleral inflammation, scleral thickness, or patient-reported pain; these parameters were assessed clinically by the treating uveitis specialists. Despite these limitations, our study contributes to the growing evidence supporting the use of anterior STI for non-infectious, non-necrotizing anterior scleritis, offering targeted anti-inflammatory therapy while reducing the need for aggressive systemic immunosuppression.

## Conclusions

STI provides an effective local treatment for non-necrotizing, non-infectious anterior scleritis, especially in patients who respond inadequately or cannot tolerate systemic therapy. This targeted approach provides rapid symptom relief, enhances patient comfort, and reduces systemic side effects. With appropriate patient selection and monitoring, it represents a safe and cost-effective adjunct in the management of scleritis.
